# 

**Published:** 1846-01

**Authors:** 


### CONTENTS

### OF NO. I.

---

### ORIGINAL COMMUNICATIONS.

### ESSAYS AND CASES.

Art. I.—Cases of Fever with Remarks. By J. R. Buck, M. D., Lecturer on the Theory and Practice of Medicine in the Louisville Summer School of Medicine. - - - - - - - - - - 1

Art. II.—Report of a Case of Narcotism from the Exhibition of Sulphate of Morphia. By Thos. Carroll, M.D., of Cincinnati, Ohio. - - - - - - 6

Art. III.—A case of Abortion, and Retention of the Placenta. By A. R. Nelson, M.D., of Rutherford county, Tennessee. - - - - - - - - - 9

Art. IV.—The Case of Dr. Abner Baker, an alleged Monomaniac, who was Executed on the 3d of October last, for killing his brother-in-law, Daniel Bates. - 11

### REVIEWS.

Art. V.—Urinary Deposits, their Diagnosis, Pathology and Therapeutical Indications. By Golding Bird, A. M., M.D., Assistant Physician to, and Lecurer on Materia Medica at, Guy's Hospital; Licentiate of the Royal College of Physicians; late President of the Westminster Medical Society; Fellow of the Linnean Society of London; Corresponding Member of the Philosophical Institute of Basle, of the Philosophical Society of St. Andrews, of the Medical Society of Hamburgh, &c., &c. - - - - - - 20

Art. V.—Elements of Pathological Anatomy; Illustrated by colored Engravings and 250 Woodcuts. By Samuel D. Gross, M.D., Professor of Surgery in the Medical Institute of Louisville; Late Professor of Pathological Anatomy in the Medical Department of the Cincinnati College; Surgeon to the Louisville Marine Hospital, &c., &c. Second Edition, thoroughly revised and greatly enlarged. - - - 28

SELECTIONS FROM AMERICAN AND FOREIGN JOURNALS

Use of Ergot of Rye. - - - - - - - 49
Peculiar Symptoms from Tape-Worm. - - - - 51
Creosote in Dysentery. - - - - - - - 52
Danger of Arsenical Injections in Subjects intended for Dissection. - - - - - - - 52
Introduction of Extract of Belladonna and Gummy Extract of Opium into the Urethra in incarcerated Inguinal Hernia. - - - - - - - - 53
Graves' Clinical Lecture. - - - - - - - 53
Placenta Prævia treated by Prof. Simpson's Method. - - 57
Turning in Arm and Shoulder Presentations. - - - 59
Turpeth Mineral in Croup. - - - - - - 61
Phenomena of Respiration. - - - - - - 66
Report on the Progress of Surgery. - - - - - 67

ORIGINAL INTELLIGENCE.

The Western Lancet, - - - - - - - - 85
The Weather. - - - - - - - - - - 89
Life and Trial of Dr. Abner Baker. - - - - 90
Introductory Lectures. - - - - - - - 90
Neill on the Nerves. - - - - - - - 91
Louisville Summer School of Medicine. - - - - 91
Facts from Correspondents. - - - - - - 92

